# Reorganization of the American Medical College

**Published:** 1910-09

**Authors:** 


### Reorganization of the American Medical College.

Following closely upon the adjournment of the Americal Medical Association comes the news that a local sectarian school has abandoned Eclecticism in favor of regular medicine. The school in question is the American Medical College of St. Louis, which, founded in 1873, is the oldest of the local colleges in point of corporate existence. The resolution which was unanimously passed by the American's board of trustees is beautiful in its terseness:

"WHEREAS, Sectarianism is not in accord with the spirit of the times; be it

"*Resolved,* That the American Medical College is, and shall be, a regular school of medicine."

Thus, within a year, two Eclectic schools have joined the regular ranks. The other example is that of the Bennett College of Eclectic Medicine and Surgery, of Chicago, which changed its name to the Bennett Medical College, in 1909; and, under the leadership of Drs. William F. Waugh and J. D. Robertson, has raised the flag of regular medicine. Within the past few weeks the Bennett College and the Illinois Medical College have consolidated under the Bennett name, and have been taken under the wing of the Ignatius Loyola University.

The American's change in policy has been favorably received not only by members of the profession but also by the lay press. Thus the *St. Louis Star,* in a scholarly editorial on "Medical Catholicity," says:

"The action of the American Medical College is a gratifying evidence of a broader and more Catholic spirit in the medical profession, which promises much in the campaign for the prevention of disease."

The newly-elected dean of the American College states that the institution will be conducted as a law-abiding, high-grade school, which will be non-sectarian and co-educational. We are informed that the annual course will be of nine months' duration—one month longer than that of any other St. Louis medical college. The *Review* trusts that success will crown the efforts of the gentlemen who have reorganized the American Medical College.—*Editorial from St. Louis Medical Review,* June, 1910.

---

For the diagnosis of fractures of the upper end of the femur careful measurements are often of greater value than any manipulations —and much safer.—*American Journal of Surgery.*

